# Peroxisome Proliferator-Activated Receptor Targets for the Treatment of Metabolic Diseases

**DOI:** 10.1155/2013/549627

**Published:** 2013-05-27

**Authors:** Francisco A. Monsalve, Radha D. Pyarasani, Fernando Delgado-Lopez, Rodrigo Moore-Carrasco

**Affiliations:** ^1^Departamento Ciencias Biomédicas, Facultad Ciencias de la Salud, Universidad de Talca, Chile; ^2^Instituto de Químicas y Recursos Naturales, Universidad de Talca, Chile; ^3^Facultad de Medicina, Universidad Católica del Maule, Chile; ^4^Departamento de Bioquímica Clínica e Inmunohematología, Facultad Ciencias de la Salud, Universidad de Talca, Chile

## Abstract

Metabolic syndrome is estimated to affect more than one in five adults, and its prevalence is growing in the adult and pediatric populations. The most widely recognized metabolic risk factors are atherogenic dyslipidemia, elevated blood pressure, and elevated plasma glucose. Individuals with these characteristics commonly manifest a prothrombotic state and a proinflammatory state as well. Peroxisome proliferator-activated receptors (PPARs) may serve as potential therapeutic targets for treating the metabolic syndrome and its related risk factors. The PPARs are transcriptional factors belonging to the ligand-activated nuclear receptor superfamily. So far, three isoforms of PPARs have been identified, namely, PPAR-**α**, PPAR-**β**/**δ**, and PPAR-**γ**. Various endogenous and exogenous ligands of PPARs have been identified. PPAR-**α** and PPAR-**γ** are mainly involved in regulating lipid metabolism, insulin sensitivity, and glucose homeostasis, and their agonists are used in the treatment of hyperlipidemia and T2DM. Whereas PPAR-**β**/**δ** function is to regulate lipid metabolism, glucose homeostasis, anti-inflammation, and fatty acid oxidation and its agonists are used in the treatment of metabolic syndrome and cardiovascular diseases. This review mainly focuses on the biological role of PPARs in gene regulation and metabolic diseases, with particular focus on the therapeutic potential of PPAR modulators in the treatment of thrombosis.

## 1. Introduction

According to World Health Organization global status reports, 80% of the 347 million people with diabetes globally will die of cardiovascular disease [1], and it will be the 7th leading cause of death in 2030 [2]. Moreover, International Diabetes Federation (IDF) predicts that people with metabolic syndrome have a fivefold greater risk of developing type 2 diabetes mellitus (T2DM). Until now a quarter of the world's adults has metabolic syndrome, and it is becoming more common due to a rise in obesity. In the future, it may overtake smoking as the leading risk factor for heart disease. To define, metabolic syndrome is a cluster of metabolic abnormalities which includes hyperlipidemia (elevated triglycerides (TG), low serum high-density lipoprotein (HDL) cholesterol), hypertension, central obesity, and elevated blood glucose [3]. However, the risk of developing metabolic syndrome is closely linked to overweight, obesity, and lack of physical activity. Furthermore, insulin resistance also may raise the risk for metabolic syndrome. Accumulating data reveals that the prevalence of this syndrome within individual cohorts varies with age, gender, and ethnicity. The pathological factors responsible for this syndrome can be high waist measurement of 35 inches or more for women or 40 inches or more for men, a high triglyceride level of 150 mg/dL or higher (The mg/dL is milligrams per deciliter—the units used to measure triglycerides, cholesterol, and blood sugar.), and a low HDL cholesterol level sometimes called as “good” cholesterol because it helps to remove cholesterol from arteries. An HDL cholesterol level of less than 50 mg/dL for women and less than 40 mg/dL for men is a risk factor, and also high blood pressure of 130/85 mmHg or (The mmHg is millimeters of mercury—the units used to measure blood pressure) high fasting blood sugar level between 100 and 125 mg/dL is considered prediabetes. A fasting blood sugar level of 126 mg/dL or higher is considered diabetes. A fasting blood sugar level of 100 mg/dL or higher (or being on medicine to treat high blood sugar) is a metabolic risk factor.

From investigations, metabolic syndrome has been found to be associated with a greater risk of coronary heart disease, stroke, diabetes, and cardiovascular mortality than the risk conferred by each one of its individual components [4]. Even though the etiology of metabolic syndrome has not yet established definitely, but growing incidences and pathetic states raise an alert to reduce the risk factors of metabolic syndrome through better lifestyle and the treatment with better therapeutics which are the cornerstones for the management of metabolic syndrome. During the last decade, it has been shown that pharmacological activations of peroxisome proliferator-activated receptors (PPARs) are effective therapeutic approaches to correct some aspects of metabolic syndrome mainly hypertriglyceridemia (fibrates) and type 2 diabetes mellitus (thiazolidinediones) [5].

## 2. Peroxisome Proliferator-Activated Receptors (PPARs)

Peroxisome proliferator-activated receptor (PPAR) is a subfamily of nuclear hormone receptors [6–8], that function as ligand-activated transcription factors to regulate various biological processes. Peroxisome proliferation was first reported in rats treated with clofibrate in the 1960s [9]. Later on, a number of compounds were discovered which share the same characteristic of inducing peroxisome proliferation. Thus, they were named as peroxisome proliferators. In 1990, the first receptor for these compounds was cloned from mouse liver and named it as peroxisome proliferator-activated receptor (PPAR) [10]. Shortly, it was realized that PPARs in fact represent a group of three receptors PPAR-*α*, PPAR-*β*/*δ*, and PPAR-*γ* [11]. All PPAR isoforms function mainly as transcription factors [12]. They control and regulate the expression of large number of genes involved in regulating the intermediary metabolism of glucose and lipids, homeostasis, adipogenesis, insulin sensitivity, immune response, cell growth, and differentiation [6, 12–15]. A variety of endogenous and synthetic ligands for PPARs have been identified [16], activation of PPARs by a suitable ligand will result in the recruitment of coactivators and loss of co-repressors that remodel chromatin and activate gene transcription. Furthermore its diverse distribution in tissue has been shown to have multiple functions upon activation such as adipogenesis, fatty acid oxidation, and anti-inflammation [17].

During the last decade it has been extensively demonstrated that risk factors of metabolic syndrome often associated with obesity, characterized by macrophage infiltration and activation in adipose tissue and liver can be treated by PPARS targets [18]. In fact, inflammation a major determinant of health complications seen in overweight and obesity, which underscores the link between nutrition, metabolic organs, and the immune system, can be regulated by PPARs targets [19–21]. This review focuses on the characterization of PPARs family, mechanism of action, ligand selectivity, and physiologic role of the PPAR family and then discusses the understanding of the pathogenic roles of metabolic syndrome and its treatment with PPARs agonists, with particular focus on the therapeutic potentials of PPAR modulators in the treatment of thrombosis.

### 2.1. Mechanisms of Action of PPARs

**Figure 1:**
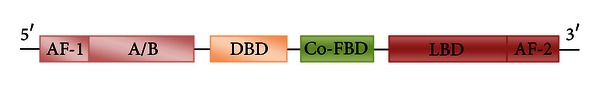
Schematic representation of the functional domains of PPARs. The PPARs are composed of four different domains. The A/B domain is located in the N-terminal region with AF-1 which is responsible for phosphorylation, C domain is involved in DNA binding domain, the D domain is the region of cofactors coupling and E/F domain is the specific domain for the ligands, associated with E/F domain is AF-2, which promotes the recruitment of cofactors necessary for the process of gene transcription.

All three isoforms of PPAR possess similar structural and functional features. Principally, four functional domains have been identified in PPARs, called A/B, C, D, and E/F (Figure 1). The N-terminal A/B domain contains a ligand-independent activation function 1 (AF-1) [22], responsible for the phosphorylation of PPAR. The DNA binding domain (DBD) or C domain promotes the binding of PPAR to the peroxisome proliferation response element (PPRE) in the promoter region of target genes [23]. The D domain or co-FBD is a site for cofactors coupling. The E/F domain or ligand-binding domain (LBD) is responsible for ligand specificity and activation of PPAR binding to the PPRE, which increases the expression of targeted genes. Recruitment of PPAR cofactors is to assist the gene transcription processes carried out by the ligand-dependent activation function 2 (AF-2), which is located in the E/F domain [24]. 

**Figure 2:**
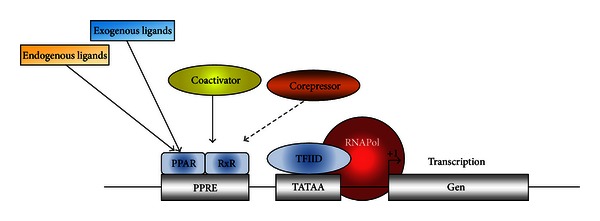
Gene transcription mechanisms of PPAR. In inactivated state, PPAR interacts with the corepressor, and this complex has Histone deacetylase activity, thus inhibiting the transcription process. After the binding of the exogenous ligand (drug) or endogenous ligand (fatty acids, prostaglandins, etc.), with the PPAR it is activated and it heterodimerizes with RXR and recruits coactivators, which have histone acetylase activity facilitating the transcription of several genes.

Substantial progress has been made to delineate the molecular mechanisms that mediate PPAR-regulated gene expression and associated cellular functions. In the classical model of PPAR activation, PPAR with RXR nuclear receptor is heterodimerized with PPRE termed DR-1, which consists of direct repeats of AGGTCA separated by a single intervening nucleotide [25, 26]. Activation of transcription through this dimer is blocked by associated corepressor proteins, such as nuclear receptor corepressors (NCoR), histone deacetylases (HDAC), and G-protein pathway suppressor 2 (GPS2) [27, 28]. The addition of ligand causes dissociation of the corepressor proteins followed by the recruitment of coactivators such as PPAR coactivator (PGC-1), the histone acetyltransferase p300, CREB binding protein (CBP), and steroid receptor coactivator (SRC)-1. Formation of the PPAR activation complex leads to histone modification (e.g., through acetylation) and altered expression of genes involved in fatty acid metabolism, lipid homeostasis, and adipocyte differentiation [29, 30]. Like PPARs, RXRs exist in three different isoforms, resulting in different combinations of heterodimers. The formation of different heterodimers seems to influence promoter recognition on the target gene sequences resulting in various metabolic processes. Furthermore, activation of PPARs by natural and synthetic ligands, other factors such as RXR, PPREs and cofactors also play a fundamental role in the process of desired transcription. The mechanisms by which activated PPARs initiate gene transcription are illustrated in Figure 2.

Recent studies of new millennium reveal new ways to activate PPARs and affect physiology. Although PPAR is commonly parked on DR-1 with RXR waiting for a ligand to activate it, there appear to be other modes of PPAR action. As with other nuclear receptors, heat shock proteins (HSP) may facilitate the folding of newly translated PPARs. The association of PPAR with Hsps, and perhaps other proteins, may keep PPAR in the cytoplasm until the appropriate ligand binds the PPAR in LBD, leading to protein dissociation and nuclear import of PPAR. In an even simpler scenario, PPAR may remain soluble, presumably in the nucleus. Ligand binding then facilitates the heterodimerization of PPAR with RXR. Another pattern for PPAR action involves its heterodimerization with a nuclear receptor other than RXR, such as the thyroid hormone receptor (THR). In these cases, the DNA pattern recognized by the heterodimer may vary from the usual DR-1 pattern. Finally, activation of PPAR by its ligand may allow it to associate with other transcription factors, such as p65 or c-jun. The binding of PPAR with p65 will prevent completion of signaling through the NF-*κ*B pathway, and binding with c-jun will interfere with AP-1 signaling pathway.

## 3. Ligands for PPAR Isoforms

From elucidated crystal structure studies of PPAR, the divergent amino acid sequence in the LBD of the three PPAR isoforms is thought to provide the molecular basis for ligand selectivity. And a large ligand-binding pocket (1300 Å) is found to exist in all three PPAR isoforms, allowing diverse and structurally distinct compounds access to the LBD [31] and enabling PPAR to sense a broad range of endogenous and exogenous compounds. A variety of endogenous and exogenous compounds, including fatty acids and their metabolites [32], industrial chemicals such as herbicides and plasticizers as well as synthetic pharmaceutical agents have been shown to bind to activate PPAR [33]. These ligand activated PPARs regulate metabolic activities leading to FA catabolism, lipid storage, glucose metabolism, cardiovascular risks and other effects, such as those affecting inflammation [34–36]. A variety of ligands, including n-3 and n-6 fatty acids (FAs), eicosanoids, and a few endocannabinoids and phospholipids, have been identified as PPAR endogenous ligands. Although many fatty acids are capable of activating all three PPAR isoforms [37, 38], some preference for specific fatty acids by each PPAR has been demonstrated.  Table 1 shows fatty acids and their derivatives, including 8-hydroxyeicosatetraenoic acid, the arachidonic acid lipoxygenase metabolite LTB4, and arachidonate monooxygenase metabolite epoxyeicosatrienoic acids which have been shown to potently activate PPAR-*α* [33, 37, 39]. Synthetic lipid-lowering drug fibrates and clofibrates are also potent ligands for activating PPAR-*α* and are clinically proved to be lipid lowering drugs [40–42]. Endogenous arachidonic acid cyclooxygenase metabolite prostacyclin, the linoleic acid 15-lipoxygenase-1 product 13-S-hydroxyoctadecadienoic acid and synthetic compounds including L-165041 and GW2433 have been found to be selective PPAR-*β*/-*δ* ligands [43, 44]. Naturally occurring PPAR-*γ* ligands including 15-deoxy-(12,14)-prostaglandin J2 [45] and oxidized metabolites of linoleic acid 9-hydroxy- and 13-hydroxy-octadecadienoic acids have been identified [46, 47]. Furthermore, synthetic antidiabetic TZD including rosiglitazone (Avandia) and pioglitazone (ACTOS) are potent PPAR-*γ* selective agonists and have been very effective in improving glycemic control via insulin sensitization (see Table 1). Interestingly, recent investigations reveal the existence of PPARs isoforms even in human platelets. This on upregulation of these PPARs inhibits platelet activation through nongenomic mechanisms [48]. Furthermore, the most crucial factor noticed is that the activity of these ligands depends on their presence in cells or tissues enriched in PPARs, their binding specificity toward the different PPARs and the availability of coregulators that can act either as coactivators or corepressors of transcription. Given the variety and anatomic distribution of endogenous PPAR ligands, and the combinations in which they occur depending on physiological (e.g., abundance and composition of food, physical activity) and pathophysiological conditions (e.g., hyperlipidemia, hypertension, diabetes, chronic inflammation, cancer, and atherosclerosis), and it is difficult to carefully evaluate the roles of each PPAR ligand in a given cell at a fixed time-point, and this remains a major challenge in the field of investigation.

But it is tempting to speculate that the diversity of PPAR functions has been acquired in association with the rich variety of ligands. With the development and clinical use of PPAR ligands in the past decade have greatly advanced our understanding of the physiological and pathophysiological roles of PPAR and therapeutic implications of modulating these receptors (see Table 1).

## 4. PPAR-*γ*

PPAR-*γ* is most widely expressed in adipose tissue but is also expressed in immune/inflammatory cells (e.g., monocytes, macrophages), mucosa of the colon and cecum, and the placenta. Its expression is the lowest in skeletal muscle and liver. PPAR-*γ* is essential for the differentiation and functioning of brown and white adipocytes and promotes the accumulation of lipids in the adipocytes [49]. There are three isoforms of PPAR-*γ* well identified, namely, PPAR-*γ*1, PPAR-*γ*2, and PPAR-*γ*3 and are derived from the same gene by the use of alternative promoters [50, 51]. PPAR-*γ*1 differs by the presence of 30 amino acids extras in the N-terminus region. PPAR-*γ*1 and *γ*3 RNA transcripts translate into the identical PPAR-*γ*1 protein. From animal studies, in knock-out mice it was evident that PPARs-*γ* is specific for adipocytes by expressing hypocellular adipocytes, developing insulin resistance in liver but not in muscles [52]. However, since PPAR-*γ* expression is tissue dependent, PPAR-*γ*1 is found in a broad range of tissues; whereas PPAR-*γ*2 is restricted to adipose tissue and PPAR-*γ*3 is abundant in macrophages, large intestine, and white adipose tissue [51–54]. Several investigations have elucidated that adipogenesis, glucose homeostasis, and lipid metabolisms are the major mechanisms of PPARs-*γ*, and it is also involved in the improvement of insulin resistance and plays a key role in inflammation and neoplastic growth [55–59]. 

### 4.1. Implication of PPAR-*γ* Agonist in Lipid Metabolism and Insulin Sensivtity

Thiazolidinediones (TZDs) are known for anti-diabetics (troglitazone, pioglitazone, ciglitazona, and englitazone) (see Table 1). These are a class of synthetic agonists that activate PPAR-*γ*, whose properties are to improve insulin resistance and lower blood glucose levels in patients with T2DM60. Even with low concentration, PPAR-*γ* it exhibits its effect in adipose tissue, liver, and muscle [60]. Several clinical studies have evaluated the efficacy of PPAR-*γ* agonists (troglitazone, pioglitazone, and rosiglitazone) in the management of insulin resistance and T2DM [61–65]. A number of studies have assessed the effects of PPAR-*γ* agonists in the prevention of onset of T2DM.

Genomics studies have shown that PPAR-*γ* is associated with several genes that affect insulin action. This receptor causes suppression of insulin action and promotes insulin resistance. In particular, PPAR-*γ* appears to be associated with genes involved in FA transport, lipid droplet formation, and TAG synthesis and breakdown. Furthermore, activation of PPAR-*γ* increases adipocyte insulin sensitivity and this may be mediated in part by direct activation of genes encoding factors of the insulin signaling pathway [66, 67]. Synthetic agonists are successful in insulin sensitivity by blocking the interaction of PPAR-*γ* with co-repressors as a result there is an increase in the accumulation of lipids in the adipose tissue and a decrease in the release of free fatty acids [68]. The benefits of TZDs are attributed to direct effects on lipid metabolism in adipose tissue and secondary effects on lipid and glucose metabolism in liver and skeletal muscle. As elucidated, TZD treatment leads to adipocyte remodeling as a result of selective preadipocyte differentiation in subcutaneous depots and apoptosis of older and larger insulin-resistant visceral adipocytes [69]. The generated new adipocytes are smaller in size and are more sensitive to insulin [70]. Furthermore activation of PPAR-*γ* by TZDs enhances lipolysis of circulating TGs and their storage in adipose tissue. In addition, there are also numerous studies that show a direct effect of these drugs on the pancreatic *β*-cells through a reduction of the lipotoxicity on the islets of pancreas, and the mechanism of action is not yet clear, but the transcriptional repressor B-cell lymphoma-6 (BCL-6) was found to play a crucial role in reducing lipotoxicity [71, 72]. However, PPAR-*γ* activation by TZDs agonists modulates the insulin signal transduction pathway by increasing the expression of intracellular proteins such as c-Cbl-associated protein (CAP), which is predominant in insulin-sensitive tissues and correlates well with insulin sensitvity [73]. Moreover, PPAR-*γ* triggers an increase in plasma concentrations of adiponectin, a hormone secreted from adipose tissue that is found at low levels in plasma of patients with T2DM. Overall functions of adiponectin are to increase FA oxidation in liver and skeletal muscle, improve insulin sensitivity in skeletal muscle and liver, and decrease glucose production in liver, resulting in decreased circulating FFAs and TG and glucose levels in liver [74]. In addition, PPAR-*γ* affects insulin sensitivity by regulating adipocyte hormones, cytokines, and proteins that are involved in insulin resistance. In fact, the TZDs agonists are well known to have a greater effect on the secretion of adipokines. Where the action of insulin is suppressed in the adipocytes and macrophages, both produce inflammatory cytokines such as TNF-*α* and IL-6, and express TLRs. Since free FA levels are elevated in obesity, their effects may be mediated by TLRs which are thought to connect metabolism to innate immunity by secretion of inflammatory cytokines and chemokines. Other adipokines are overregulated, particularly adiponectin and resistin which are known to be potential insulin sensitizers in liver and skeletal muscle [75, 76]. 

 A recent study showed that metabolic syndrome is associated with an increased risk of all-cause cancer mortality in men [77]. A possible explanation for the increased cancer risk connected to metabolic syndrome, elevated nocturnal free-fatty acids and hyperinsulinemia, which were found in obese animals [78]. Additional pathophysiological mechanisms, which link metabolic syndrome to cancer, are elevated adipokines levels, IGF, the mitogenic action of insulin, and increased levels of reactive oxygen species [79]. This pathological state can be regulated with TZDs agonists (see Table 1).

### 4.2. Implication of PPAR-*γ* in Glucose Metabolism

 As well known insulin is the most potent physiological anabolic agent, promoting the storage and synthesis of lipids, protein, and carbohydrates and inhibiting their breakdown and release into the circulation [80]. The first step by which insulin increases energy storage or utilization involves the regulated transport of glucose into the cell, mediated by the facilitative glucose transporter Glut4. Insulin increases glucose uptake mainly by enriching the concentration of Glut4 proteins at the plasma membrane, rather than by increasing the intrinsic activity of the transporter [81, 82]. PPAR-*γ* activation by rosiglitazones was found to increase the expression and translocation of the glucose transporters GLUT1 and GLUT4 to the cell surface, thus increasing glucose uptake in adipocytes and muscle cells and reducing glucose plasma levels [83, 84]. Activation of PPAR-*γ* by TZDs decreases glycated hemoglobin (HbA1c) and fasting and postprandial glucose and lowers circulating insulin levels in patients with T2DM, largely as a consequence of the improvement in insulin sensitivity. Furthermore, TZDs stimulate the use of glycerol for TG production, thereby reducing FFA release from adipocytes and this reduction in FFAs alleviates lipotoxicity in skeletal muscle, liver, and pancreas, leading to a reduction in hepatic glucose production and improved glucose utilization in skeletal muscles, resulting in the hypoglycemic effects of TZDs. The changes in glucose homeostasis can also be partly attributed to a direct action of PPAR-*γ* activation on insulin-stimulated glucose disposal. Moreover, T2DM is associated with a state of chronic inflammation of fat cells that secrete elevated levels of cytokines; agonists of PPAR-*γ* were shown to inhibit the expression of cytokines such as resistin, tumor necrosis factor a (TNF*α*), and interleukin-6 which promote insulin resistance. PPAR-*γ* agonists trigger an increase in plasma concentrations of adiponectin, a hormone secreted from adipose tissue that is found at low levels in plasma of patients with T2DM. Adiponectin increases FA oxidation in liver and skeletal muscle. Overall, adiponectin improves insulin sensitivity in skeletal muscle and liver and decreases glucose production in liver, resulting in decreased circulating FFAs and TG and glucose levels [85]. 

### 4.3. Implications of PPAR-*γ* in Atherosclerosis and Inflammation

Interestingly PPAR-*γ* was the first reported to undergo agonist-dependent simulation, which promotes binding to nuclear receptor co-repressor-1 protein (NCoR) and stabilizes association with promoter-bound NF-*κ*B, thereby leading to the transrepression of inflammatory genes in macrophages [86–88]. Other beneficial and inhibitory effects of PPAR-*γ* agonists on inflammation were reduction in the production of proinflammatory molecules in T lymphocytes, promotion of the expression of anti-inflammatory mediators in the innate immune system, reduce cytokines (TNF-*α*, IL-1, and IL-6) production by inhibition of genes encoding pro-inflammatory molecules, and reduction of transcriptional activities Nuclear Factor-*κ*B (NF-*κ*B), AP-1, and STAT [89, 90]. PPAR-*γ* also reduces vascular smooth muscle cell proliferation, increases monocyte apoptosis, and suppresses metalloproteinase-9 expression in atherotic plaques [91–94]. During atherogenesis PPAR-*γ* is expressed by monocytes and macrophages, a strategy behind inflammation homeostasis. First, monocytes are attracted to the vascular wall of large arteries by adhering to them through integrins l and integrins endothelial cell activation. Monocytes infiltrate to the subendothelial space by a chemokine gradients, such as IL-8, which originates from the source of infection, where they will be differentiated to macrophages. This key step is already altered in response to activation of PPAR-*γ*. Thus, troglitazone (TZD) inhibits the early formation of injury atherosclerotic, decreasing the accumulation of macrophages in the intimate through the inhibition of monocyte transendothelial migration. In addition, PPAR-*γ* agonists may indirectly suppress systemic production of a proinflammatory milieu mainly by inhibiting TNF-*α*, plasminogen activator inhibitor-1, and IL-6 expression in adipose tissue [95, 96]. Elevated levels of HDL cholesterol and reduced triglyceride levels may also contribute to the beneficial effect of PPAR-*γ* agonists in atherosclerosis [97]. On the basis of these data, it seems likely that TZD PPAR-*γ* agonists will have beneficial effects on atherosclerosis and provide a promising therapy for the metabolic syndrome and its cardiovascular complications.

Novel antagonist and partial agonists of PPAR-*γ* have recently been identified; triterpenoid 2-cyano-3,12-dioxooleana-1,9-dien-28-oic acid (CDDO) is a partial agonist with anti-inflammatory properties [98] and bisphenol diglycidyl ether (BADGE) and LG-100641 have been identified as antagonists for PPAR-*γ* (see Table 1) [99, 100]. Even though these compounds have little clinical significance, they can be used to understand the physiology of the PPAR-*γ* and for the identification of new ligands. In addition to synthetic chemical methods, research in natural products has also yielded potent PPAR-*γ* agonists from several medicinal plants. Saurufuran A from *Saururus chinensis* (Saururaceae) [101], flavonoids such as chrysin, apigenin and kaempferol [102], and phenolic compounds from *Glycyrrhiza uralensis* (Fabaceae) [103] are recently identified PPAR-*γ* agonists to treat risk factors of metabolic syndrome.

## 5. PPAR-*α*

PPAR-*α* belongs to the nuclear receptor superfamily and was the first to be identified as PPAR receptor. It was named based on the ability to be activated by substances leading to the proliferation of peroxisomes in rodents. PPAR-*α* is expressed in numerous tissues, both in rodents and in humans, for example, in liver, kidneys, heart, skeletal muscle, and Brown fat [104, 105], and in a wide range of vascular cells such as endothelial cells, vascular smooth muscle cells (VSMCs), and monocytes/macrophages [106–109]. Accumulating evidence demonstrates that PPAR-*α* is an important modulator of the metabolic syndrome and may be a therapeutic target for treating some of its features, especially cardiovascular complications. PPAR-*α* has been identified as a key regulator of the genes involved in fatty acid oxidation, which occurs in mitochondria, peroxisomes, and microsomes in the liver [110]. Transcription and protein levels of critical enzymes in *β*-oxidation and *ω*-oxidation pathways are direct targets of PPAR-*α*, including acyl CoA oxidase, carnitine palmitoyl transferase I, mitochondrial hydroxymethylglutaryl CoA synthase, and cytochrome P450 4A enzymes (CYP4A) [111]. By increasing the expression of these genes, PPAR-*α* ligands significantly activate hepatic fatty acid oxidation, whereas genetic inactivation of the PPAR-*α* gene results in massive accumulation of lipids in the liver, severe hypoketonemia, hypoglycemia, hypothermia and elevated plasma free fatty acid levels [112]. These data clearly indicate that PPAR-*α* is a key factor in governing metabolic adaptation to increased fatty acids. PPAR-*α* is also known to regulate the expression of genes for the transport of proteins and enzymes that are involved in the processes of inflammation. Many lines of study show that PPAR-*α* regulates lipid homeostasis. By increasing *β*-oxidation of fatty acids and providing energy to the cell, it also cuts the long-chain fatty acids, thereby preventing the accumulation and toxicity of lipids in cells [113]. In addition, they also stimulate the cellular uptake of fatty acids by increasing the expression of the fatty acid transport protein (FATP) and fatty acid translocase (FAT) [114]. 

### 5.1. Implication of PPAR-*α* in Lipid Metabolism

PPAR-*α* plays a critical role in lipid metabolism. Its known target genes are involved in almost all aspects of lipid metabolism, including uptake, binding, and oxidation of fatty acids; lipoprotein assembly; and lipid transport [115, 116]. Synthetic PPAR-*α* ligands, such as gemfibrozil, fenofibrate, and clofibrate (see Table 1), have been used in clinical practice as hypolipidemic agents for more than 3 decades. Notably, activation of PPAR-*α* with synthetic agonists not only increases hepatic FAO, but it also increases lipogenesis and FA chain elongation, in a sterol regulatory element binding protein (SREBP)-1c dependent manner. This indicates that PPAR-*α* induces the entire FA handling regimen in liver to either catabolize it or store it, thereby diminishing cytotoxic effects of free FA. As evident PPAR-*α* is highly expressed in liver and activation of this receptor promotes the expression of cytochrome P4504A (CYP4A). The CYP4A is a subclass of cytochrome P450 enzyme that catalyzes the *ω*-hydroxylation of fatty acids [111] which is beneficial in reducing the synthesis of triglycerides (TGs). In addition, PPAR-*α* agonists are used for the treatment of dyslipidemia, which is characterized by decreased triglycerides levels and increased HDL-c levels in plasma [117]. This state can be achieved by increasing the production of the major component of HDL-c, called Apolipoprotein A-I and A-II (APO A-I & APO A-II) [118, 119] which plays a vital role in reverse cholesterol transport (RCT) pathway from peripheral cells. Moreover, PPAR-*α* PPAR-activation further decreases TG levels by amplifying the expression of lipoprotein lipase (LPL) [120] and inhibiting APO C-III in the liver. The biological mechanism of PPAR-*α* is activated under nutrient-deficient conditions and is necessary for the process of ketogenesis, a key adaptive response to prolonged fasting. In an experiment that pharmacologically blocked transport of long-chain fatty acids into mitochondria, knockout mice developed hepatic steatosis, severe hypoketonemia, hypoglycemia, and hypertriglyceridemia [121]. This indicates that genetic inactivation of PPAR-*α* gene results in massive accumulation of lipids in the liver.

### 5.2. Implication of PPAR-*α* in Glucose Metabolism and Insulin Sensitivity

Metformin is most commonly used in the initial management of T2DM, but its mechanisms by which it lowers glucose levels are not completely known. T2DM patients with metformin treatment reduce the production of hepatic glucose via gluconeogenesis decrease. It is believed that metformin exerts its action via incretins, increasing levels of GLP-1 (glucagon-like peptides) via PPAR-*α* dependent or independent mechanism [122]. Altered PPAR-*α* has also been implicated in the pathogenesis of obesity and insulin resistance [123]. Activation of PPAR-*α* reduces weight gain in rodents, and inactivation of PPAR-*α* results in a late onset of obese phenotype [124, 125]. Treatment of PPAR-*α* null mice with a high-fat diet leads to a more dramatic increase in body weight [126], further suggesting that PPAR-*α* may be involved in the genesis of obesity. Evidence has recently emerged suggesting that PPAR-*α* is an important regulator of insulin sensitivity. Treatment with PPAR-*α* activators dramatically improved insulin resistance and glycemic control in type 2 diabetic db/db mice and OLETF rats [127–129]. Similarly, the PPAR-*α* agonist bezafibrate markedly improved glucose intolerance and insulin resistance in a lipoatrophic diabetic patient [130]. More importantly, recently it was reported that the PPAR-*α* agonist fenofibrate prevents the development of diabetes in insulin-resistant obese OLETF rats. Although the downstream mechanisms underlying these observations are not clear, they are consistent with the idea that PPAR-*α* plays a critical role in regulating insulin sensitivity *in vivo *and that its activation may lead to the delay of onset of type 2 diabetes [128]. 

### 5.3. Atherosclerotic Lesions Effect and Myocardial Regulation

 Activation of PPAR-*α* in endothelial cell receptor will block the cellular adhesion induced by cytokines and increase the expression of the HDL in CLA-1/SR-BI receptor [131] and the cholesterol efflux pump ATP binding cassette A-1 (ABCA1) transporter in macrophages [132]. Some studies suggest that activation of PPAR-*α* increased the expression of ABCA1 by enhancing the expression of the liver X receptor, LXR-*α* [133]. Thus, PPAR-*α* and LXR-*α* agonists may induce positive effects on atherosclerotic lesions. Finally, fibrate PPAR-*α* activators have been reported to potently reduce atherosclerosis both in apoE^−/−^ mice and in human ApoAI transgenic apoE^−/−^ mice [134]. In heart, PPAR-*α* supplies energy to the myocardium by regulating the genes responsible for fatty acid uptake and *β*-oxidation [135]. This function is achieved by decreasing fatty acid oxidation and inhibiting lipoprotein lipase (LPL) [136]. Consequently all these mechanisms have been reducing progression of atherosclerosis and decreasing the incidence of coronary events in several clinical studies. More important, fibrate treatment of patients who exhibit more than three features characteristic of the metabolic syndrome (diabetes, glucose intolerance or high fasting insulin, hypertension, obesity and high triglycerides or low HDL cholesterol) was associated with a significant 35% risk reduction in the rate of coronary artery disease death, nonfatal myocardial infarction, or definite stroke [137–139]. These data support the concept that fibrate PPAR-*α* agonists may be particularly effective agents for the cardiovascular complications of the metabolic syndrome.

### 5.4. Implication of PPAR-*α* in Anti-Inflammation

PPAR-*α* agonist's exhibit anti-inflammatory effect in vascular cells by inhibiting the production of some inflammatory cytokines such as TNF-*α*, IL-6 in blood, and decreased the expression of cyclooxygenase-2 in VSMCs by NF-*κ*B signaling repression in cardiac myositis [140] and induced expression of VACAM-1 [141] and increased expression of endothelial nitric oxide synthase (eNOS) [142]. Moreover, WY14.643, a potent agonist of PPAR-*α*, can directly increase the expression of adiponectins, antidiabetics, antiatherosclerosis, and anti-inflammatory effects [143]. 

## 6. PPAR-*β*/-*δ*

PPAR-*β*/-*δ* has important functions in the skin, gut, placenta, skeletal and heart muscles, adipose tissue, and brain [144]. Contrary to PPAR-*α* and PPAR-*γ*, PPAR-*δ* is expressed all over the body, but its pharmacology is less understood than the other subtypes [145]. It is the most abundant isoform among the three PPARs in skeletal muscle. Since skeletal muscle accounts for about 50% of whole body mass, and more than 50% of metabolism occurs in it. Therefore, activities involved in muscle contraction may significantly increase energy expenditure and result in the usage of glucose or breakdown of fat as fuel. PPAR-*δ* encourages skeletal muscle to burn stored fat as fuel. It can be greatly beneficial because it decreases triglycerides and LDL-cholesterol (bad cholesterol) levels and increases insulin sensitivity and HDL-cholesterol (good cholesterol) levels. Since metabolic syndrome is a problem caused by too much fat stored in the body, PPAR-*δ* has been recognized to be a possible solution because it makes the body burn more fat. Fat is the main source of fuel for endurance exercise; therefore, PPAR-*δ* is produced for increasing the breakdown of body fat to generate energy. Indeed, the increase of PPAR-*δ* helps to lose fat and exercise longer because it facilitates the use of fat. So far reported candidates for endogenous activators of PPAR-*β*/-*δ* are fatty acids, triglycerides, and prostacyclin. In addition a number of synthetic compounds including L-165,041, GW501516, GW0742, and GW610742 (see Table 1) [144, 146–149] have also been designed as selective PPAR-*β*/-*δ* ligands. Unlike PPAR-*α* (fibrates) or -*γ*, (glitazones) there are no PPAR-*β*/-*δ* drugs in clinical use yet, though lead compounds such as GW501516 are in phase II clinical trials for dyslipidaemia. As yet only one selective PPAR-*β*/-*δ* antagonist has been described GSK0660. In skeletal muscle myoblast cells in culture, GSK0660 inhibits GW0742 induction of established PPAR-*β*/-*δ* target genes (carnitine palmitoyltransferase 1A, angiopoietin-like 4 protein, and pyruvate dehydrogenase kinase-4), along with the concurrent PPAR-*β*/-*δ* induced increase in fatty acid oxidation [150]. As yet this is the only report on GSK0660, so still nothing is known regarding its long-term effects *in vivo*.

### 6.1. Implication of PPAR-*β*/*δ* in the Regulation of Lipid Metabolism

PPAR-*β*/-*δ* is the most abundant isoform among the three PPARs in skeletal muscle; it acts as a central regulator of fatty acid catabolism in skeletal muscle by controlling expression of proteins directly implicated in this metabolic pathway and also by increasing the intrinsic oxidative capability of the tissue. Since skeletal muscle accounts for about 50% of whole body mass and more than 50% of metabolism occurs in it. Therefore, activities involved in muscle contraction may significantly increase energy expenditure and result in the usage of glucose or breakdown of fat as fuel. PPAR-*β*/-*δ* encourages skeletal muscle to burn stored fat as fuel. And this is greatly beneficial because it decreases triglycerides and LDL-cholesterol (bad cholesterol) levels and increases insulin sensitivity and HDL-cholesterol (good cholesterol) levels. Since metabolic syndrome is a problem caused by too much fat stored in the body, PPAR-*β*/-*δ* has been recognized to be a possible solution because it makes the body burn more fat. Since fat is the main source of fuel for endurance exercise; therefore, PPAR-*β*/-*δ* is produced for increasing the breakdown of body fat to generate energy. Indeed, the increase of PPAR-*β*/-*δ* helps to lose fat and exercise longer because it facilitates the use of fat. Synthetic PPAR-*β*/-*δ* ligands are considered as effective compounds to improve metabolic syndrome. An experimental study using obese diabetic db/db mice as a model was examined to see the effect of a PPAR-*β*/-*δ* specific agonist L-165041 on plasma lipid profile [151]. L-165041 treatment significantly increases HDL cholesterol levels, possibly associated with a decreased lipoprotein lipase activity in the white adipose tissue. Confirmatory results were recently obtained with a more potent and selective PPAR-*β*/-*δ* agonist, GW501516 (Ki = 1.1 ± 0.1 nM) in insulin-resistant middle-aged obese rhesus monkeys [152]. These results showed that GW501516 causes a dramatic dose dependent increase in serum HDL cholesterol and a reduction in LDL and triglyceride, suggesting that activation of PPAR-*β*/-*δ* is associated with a less atherogenic lipid profile and antidiabetic action [152–154]. PPAR-*β*/-*δ* has also exhibited a potential role in placentation, adiposity, colorectal cancer, and diabetic factors. GW0742 is a closely related analog of GW501516 and shows equivalent potency and selectivity for PPAR-*β*/-*δ* [152].

### 6.2. Implication of PPAR-*δ* in the Regulation of Insulin Sensitivity

The role of PPAR-*β*/-*δ* in the regulation of glucose homeostasis has emerged with the findings that PPAR-*β*/-*δ* agonists reduce adiposity and improve glucose tolerance and insulin sensitivity in different mouse models of obesity [153]. In addition, PPAR-*β*/-*δ* null mice display glucose intolerance when fed a chow diet. GW501516 treatment reduced insulin levels in obese insulin-resistant monkeys [152]. The use of PPAR-*β*/-*δ* tissue-specific transgenic mice, in adipose tissue or skeletal muscle, has shown that activated PPAR-*β*/-*δ* induces the expression of genes involved in FA oxidation and in energy expenditure through the induction of uncoupling proteins in brown adipose tissue and in skeletal muscle [154–156]. As a consequence, substrate supply for lipid storage in white adipose tissue is decreased, resulting in the reduction of adiposity. It is also believed that PPAR-*β*/-*δ* induces fat burning in muscle, which together with an overall improvement in systemic lipid metabolism is responsible for lowering fat overload in insulin-sensitive tissues, thereby reducing insulin resistance. 

In a primate model of the metabolic syndrome, the PPAR-*β*/-*δ* selective agonist GW501516 dose-dependently lowered plasma insulin levels, without adverse effects on glycemic control [152]. Similarly, in ob/ob mice, a model of the metabolic syndrome, a PPAR-*β*/-*δ* specific agonist markedly improved glucose tolerance and insulin resistance [153]. Although the underlying mechanism is unclear, activation of PPAR-*β*/-*δ* in skeletal muscle, which has a significant role in insulin sensitivity, has been proposed to account for the beneficial metabolic effects of PPAR-*β*/-*δ* agonists on lipid profile and insulin resistance, possibly as a result of increased fatty acid catabolism, cholesterol efflux, energy expenditure [153–157], and oxidative capability [155] in the muscle. Recently it was described that lipid peroxidation and the consequent production of 4-HNE in *β*-cells stimulate the secretion of insulin via a dependent mechanism, and these results were confirmed by treating these cells with an antagonist of this nuclear factor that resulted in blocking of the effect [158]. Activation of PPAR-*β*/-*δ* with agonist prevents induction of the transcription factor STAT3 by inhibiting the activation of ERK and inhibiting the interaction of STAT3 and Hsp90; this translates into the prevention of insulin resistance in adipose tissue [159]. In PPAR-*β*/-*δ* knockout mice it showed an obese phenotype when they were fed a diet rich in fats. The overexpression of PPAR-*β*/-*δ* or activation by the ligand GW501516 shows induction of muscle fiber type I, cell type rich in mitochondria that allow mice to undertake large periods of aerobic activity, and for this reason they are called mouse marathon runner. These mice were also resistant to diet-induced obesity and insulin resistance [160]. Finally, with the availability of three synthetic ligands (GW501516, GW0742, and L-165041) that activate PPAR-*β*/-*δ* at very low concentrations both *in vivo* and *in vitro* with high selectivity over other PPAR isotypes [161] had led to a huge increase in experimental studies on the role of PPAR-*β*/-*δ* in cellular processes. The IC_50_ for these compounds assessed with recombinant human PPAR-*β*/-*δ* were 1.0 nM for GW0742, 1.1 nM for GW501516, and 50 nM for L-165041 [161, 162].

### 6.3. Implication of PPAR-*β*/*δ* in Regulating Cardiovascular Complications and Atherosclerotic Lesions

**Figure 3:**
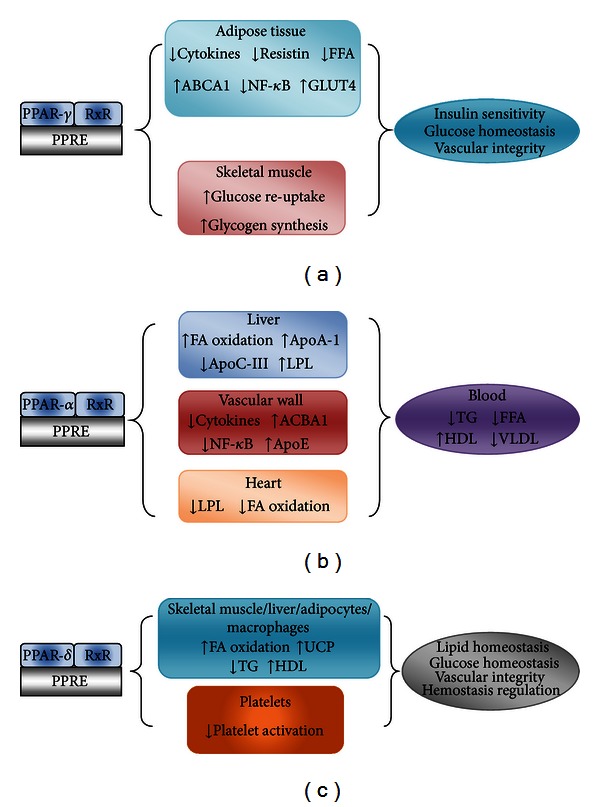
Mechanism of transcription and biological effects in different organs. (a) PPAR-*γ* exhibits anti-diabetic and atherosclerotic effects in adipocytes and skeletal muscle. (b) PPAR-*α* has multiple effects on liver, heart, and vascular wall. (c) PPAR-*δ* is expressed widely throughout the body and its gene expression is involved with the metabolism of lipids and glucose, as well as in decreasing the platelet activation. (Modified from [87].)

Chronic low-grade inflammation plays a role in cardiac hypertrophy and heart failure [163]. Ongoing basic studies have demonstrated the role of PPAR-*β*/-*δ* in ameliorating cardiovascular complications. Few studies have shown that PPAR-*β*/-*δ* ligands have the potential to inhibit cardiac hypertrophy due to their inhibitory activity on NF-*κ*B transcription factor which produces inflammatory cytokines such as TNF-*α*, MCP-1, and IL-6, and these are secreted by cardiac cells under various pathophysiological stimuli which may participate in myocardial inflammation [163]. Activated PPAR-*β*/-*δ* also dampens LPS-induced TNF-*α* inflammation signaling in cultured cardiomyocytes and blocks palmitate-induced inflammatory pathways in mouse heart and human cardiac cells through protein-protein interaction between PPAR-*β*/-*δ* and p65, suggesting inhibition of NF-*κ*B [164, 165]. From these biological effects (Figure 3), PPAR-*β*/-*δ* may serve as a potential therapeutic target to prevent cardiac hypertrophy and heart failure in metabolic disorders. PPAR-*β*/-*δ* also attenuates progressive cardiac fibrosis occurring in diabetic cardiomyopathy. In null mice, PPAR-*β*/-*δ* specific cardiomyocyte and macrophage were infused with angiotensin II to trigger cardiac fibrosis, and then treated with pioglitazone; it is observed that the macrophage and not cardiomyocyte PPAR-*β*/-*δ* that attenuates fibrosis [156]. 

Finally, PPAR-*β*/-*δ* has recently been proposed as a potential target for modulating foam cell and macrophage activation in atherosclerosis. *In vitro *studies suggested that PPAR-*β*/-*δ* activation in cultured macrophage results in increased expression of the reverse cholesterol transporter ATP-binding cassette A1 and enhances efflux of cholesterol [152]. PPAR-*β*/-*δ* also participates in cellular VLDL sensing and mediates VLDL-triglyceride-driven transcription events in macrophage [157]. VLDL-triglyceride treatment results in triglyceride accumulation and the induction of adipocyte phenotype, which can be blocked by disruption of the PPAR-*β*/-*δ* gene. Furthermore PPAR-*β*/-*δ* also attenuates atherogenic inflammation. Its synthetic ligands GW0742 and GW501516 reduce atherosclerosis in low-density lipoprotein receptor (LDLR) null mice, possibly by decreasing monocyte chemotactic protein-1 (MCP-1), intercellular adhesion molecule-1, and TNF-*α* expression [166, 167]. These new observations suggest that agonists for PAR-*β*/-*δ* may be effective agents to reverse cholesterol deposition in foam cells in atherotic lesions and therefore decrease cardiovascular disease associated with the metabolic syndrome. Taken together, PPAR-*β*/-*δ* is a critical player in the pathogenesis of the metabolic syndrome, and its ligands may provide useful agents for treating dyslipidemia, obesity, insulin resistance, and atherosclerosis.

## 7. Other Biological Mechanisms

The role of PPAR ligands has been well established in some very important therapeutic areas such as diabetes, obesity, cardiovascular diseases, and inflammation. But, more recently, it is becoming clear that they are also involved in antithrombosis. Diabetes mellitus is associated with a heightened risk of developing atherosclerotic vascular disease and its acute thrombotic complications, such as myocardial infarction [168]. Interestingly platelets play an important role in hemostasis and thrombosis, and there is an increasing evidence showing they are involved in mechanisms of inflammation and host defense, contributing to the pathogenesis and progression of vascular complications in the T2DM [169]. Therefore, treatment with antiplatelet agents may be a beneficial strategy to prevent and improve thrombosis-related cardiovascular diseases [170]. 

Platelets are enucleated cells derived from megakaryocytes; they contain transcription factors, notably the peroxisome proliferator-activated receptors (PPARs). So far, three PPAR isoforms (PPAR-*α*, PPAR-*β*/-*δ*, and PPAR-*γ*) have been found in human platelets, and upregulation of PPARs inhibits platelet activation through a nongenomic mechanism [171]. Platelet activation is associated with signaling that affects cell shape and spreading, secretion, and the release of multiple prothrombotic factors; all through the binding of plasma fibrinogen and von Willebrand factor (VWF) to integrin *α*_IIb_ *β*_3_, this leads to the formation of a stable platelet thrombus [172–174]. Many findings suggest that agents with PPARs-activating effect may exert an anti-platelet activity. A recent study was done by Ching-Yu Shih and group to prove that PPARs-mediated pathways contribute to the anti-platelet activity [175]. They used a natural product, magnolol, extracted from Chinese medicinal herbs, which has demonstrated multiple pharmacological effects, including antiatherosclerosis, antioxidative, anti-inflammatory, and anti-bacterial, even anti-platelet activity [176, 177]. Magnolol is a PPAR-*γ* agonist through direct binding to the PPAR-*γ* ligand binding domain it exhibit the anti-platelet activity and inhibits various important mediator formation and signaling pathways related to platelet activation. In the presence of selective PPAR-*β* antagonist (GSK0660) or PPAR-*γ* antagonist (GW9662), the inhibition of magnolol on collagen-induced platelet aggregation and intracellular Ca^2+^ mobilization was significantly reversed. These show that the excellent anti-platelet and antithrombotic activities of magnolol are modulated by upregulation of PPAR-*β*/-*γ*-dependent pathways [178]. 

Other findings of anti-platelet activity are lipid-lowering agents such as fibrates and statins that reduce thrombotic and cardiovascular risk. These are hypolipidemic drugs which decrease cardiac events in individuals without raised levels of cholesterol. In platelets, PKC_*α*_ activation facilitates platelet aggregation [179, 180]. Simvastatin and fenofibrate drugs inhibit platelet activation by inhibiting PKC_*α*_ which are associated with PPARs. Selective PPAR-*γ* antagonist GW9662, and PPAR-*α* antagonist GW6471 showed inhibition on effects of simvastatin and fenofibrate on platelet function which are mediated by PPAR-*γ* and PPAR-*α*, respectively, and the aggregation effects of *α*-lipoic acid can be attributed to the activation of PPAR-*α*/-*γ* [172]. In another study they demonstrate the ability of PPAR-*γ* ligands to modulate collagen stimulated platelet function and suppress activation of the glycoprotein VI (GPVI) signaling pathway. PPAR-*γ* ligands inhibited collagen-stimulated platelet aggregation that was accompanied by a reduction in intracellular calcium mobilization and P-selectin exposure. PPAR-*γ* ligands inhibited thrombus formation under arterial flow conditions. The incorporation of GW9662 antagonists reversed the inhibitory actions of PPAR-*γ* agonists, implicating PPAR-*γ* as modulator. Furthermore, PPAR-*γ* ligands were found to inhibit tyrosine phosphorylation levels of multiple components of the GPVI signaling pathway. PPAR-*γ* was also found to associate with Syk and LAT after platelet activation. All this association was prevented by PPAR-*γ* agonists, indicating a potential mechanism for PPAR-*γ* function in collagen-stimulated platelet activation [181]. 

## 8. Conclusions and Future Prospects

Treatment and prevention of metabolic syndrome require lifestyle changes, including weight reduction, increased physical activity, and better diet. However, as many patients cannot control the pathology with lifestyle modification, there is a need for drugs to manage the metabolic syndrome. As discussed in this paper, that PPARs are transcription regulators which are involved in different metabolic pathways, they are able to interfere with many normal cellular processes as well as in altered processes that ultimately lead to pathology. This nuclear hormone receptor is believed to originate from a common ancestral receptor of 600 million years old. Furthermore it has managed to differentiate into several subtypes that can adapt to different ligands [182]. Its large capacity and strict control location allow these nuclear regulators to control complex processes such as inflammation or control energy homeostasis [183]. They are also attributed for their ability to regulate cellular differentiation in numerous cell lines such as keratinocytes, adipocytes, or immune system cells [182, 183]. Another factor which makes them particularly interesting is its ability to be activated or repressed by internal or natural ligands and synthetic or exogenous ligand; this capability opens thousands of possibilities in the treatment of various pathologies by numerous processes that are capable of controlling genes [184–186]. 

The pharmacological race generated new and increasingly potent agonists or antagonists, and it has prompted pharmaceutical companies to invest large sums of money on science, but nothing had been positive; unfortunately adverse effects have been reported for many synthetic compounds. For instance, one of the most important problems of PPAR agonists is cardiac toxicity which changes in the QT segment of the ECG, although the results obtained for this were controversially fair. Now recently it was confirmed that therapeutic concentrations of aleglitazar (dual agonist) show no evidence of changes in the QT segment of ECG. Consequently, the new dual agonist of PPAR alpha and gamma shows great advantages over its predecessors because the results obtained in phase I and II show a clear balance between patient safety and efficacy in improving metabolic parameters [187–191] and are extremely encouraging to start phase III. Clinical and basic sciences are on a quest to find double or triple agonists (PAN) in which the unwanted effects of agonists can be decreased significantly, and as a result individuals get a better quality of life with more potent drugs that result in increasingly lower doses (IC_50_ under toxic levels). Furthermore, with the development of PPAR delta targets, it has been shown that this nuclear regulator has become a therapeutic target in combating cardiovascular diseases that is more common in industrialized countries. PPAR delta targets will regulate the level of lipid oxidation in skeletal muscle and regulate the ratio of LDL and high density lipids [192, 193]; it is even able to inhibit the formation of foam cells induced by the small, dense LDL [194]. Recent studies have used GW501516 (a potent agonist of PPAR delta) which show a change in the lipoprotein profile, and highly atherogenic profile was passed to onefold less, proving to be a powerful cardiovascular protector for individuals with MS [193]. It is evident that platelets are extremely important in ischemic cardiovascular diseases, and it has been demonstrated that the greater number of active circulating platelets or a greater number of platelet-leukocyte complexes predict larger plates with greater lipid accumulation [195]. This fact evidences that rosiglitazone is capable of reducing the amount of circulating activated platelets and makes these compounds an ideal therapeutic target to control atherogenesis [196–198]. Above all it is tempting to speculate that the diversity of PPAR functions has been acquired in association with the rich variety of ligands. With the development and clinical use of PPAR ligands in the past decade have greatly advanced our understanding of the physiological and pathophysiological roles of PPAR and therapeutic implications of modulating these receptors. Surely the solution to metabolic diseases can be found in a drug response with super powers or with a combination of drugs that individually present positive effects and enhance the whole colud open Pandora's box, which could result in a real treatment.

## Figures and Tables

**Table 1:** Potential targets of PPARs for prevention and treatment of metabolic syndrome.

Ligands	Receptor	Selective	Function
Saturated Fatty acid	PPAR-*α* (liver, adipose tissue, kidney, heart, skeletal, muscle, large intestine)	Endogenous lipid with PPAR-*α* agonist active	Lipid and glucose metabolism
Unsaturated Fatty acid		Endogenous lipid with PPAR-*α* agonist active	Lipid and glucose metabolism
CP775146		Selective, high affinity PPAR-*α* agonist	Lipid and glucose metabolism
Fenofibrate		PPAR-*α* agonist	Treatment of hypertriglyceridemia and dyslipidemia
GW7647		Highly selective, potent PPAR-*α* agonist. Orally active	Lipid homeostasis, beta oxidation
Oleylethanolamide		PPAR-*α* agonist	Lowers body weight and hyperlipidemia
Palmitoylethanolamide		Endogenous lipid with PPAR-*α* agonist active	Anti-inflammatory, reduces pain
WY14643		Selective PPAR-*α* agonist	Atherosclerosis and anti-inflammation and prevent hyperinsulinemia
GW6471		Selective PPAR-*α* antagonist	Antagonist fenofibrates
MK886		Selective PPAR-*α* antagonist	Inhibit PPAR-*α*, *β*, *γ* activities

Fatty acids (i) Docahexanoic acid (ii) Arachidonic acid (iii) Linolenic acid	PPAR-*β* (Ubiquitous)	Endogenous lipid with PPAR-*β* agonist active	Anti-inflammation
GW501516		Highly selective, potent PPAR-*β* agonist	Increases serum HDL-c in atherogenic dyslipidemia and decreases fasting blood sugar.
GW0742		Potent PPAR-*β* agonist	Anti-inflammatory
L-165,041		PPAR-*β* selective agonist	Hyperlipidemia, hyperglicemia, ateroesclerosis, and obesity
GW610742		PPAR-*β* selective agonist	Treatment diabetic and nephropathy
FH535		PPAR-*β* selective antagonist	Treatment diabetic and nephropathy
GSK0660		PPAR-*β* selective antagonist	Inverse agonist PPAR-*β*/*γ*
GSK3787		Potent and selective PPAR-*β* antagonist	Inverse agonist PPAR-*β*/*γ*
NSAIDs		PPAR-*β* selective antagonist	Cancer

Linolenic acid	PPAR-*γ* (adipose tissue, lymphoid tissue, colon, liver, heart)	Endogenous lipid with PPAR-*γ* agonist active	Anti-inflammation
Arachidonic acid		Endogenous lipid with PPAR-*γ* agonist active	Anti-inflammation
15d-PGJ2		Endogenous lipid with PPAR-*γ* agonist active	Anti-inflammation
9-HODE		Endogenous lipid with PPAR-*γ* agonist active	Anti-inflammation
13-HODE		Endogenous lipid with PPAR-*γ* agonist active	Anti-inflammation
15-HETE		Endogenous lipid with PPAR-*γ* agonist active	Anti-inflammation
Ciglitazone		Selective PPAR-*γ* agonist	Inhibits cell proliferation
GW1929 hydrochloride		Selective PPAR-*γ*. Orally active	Decreases glucose, fatty acids, and triglyceride
LG100754		PXR:PPAR agonist	Sensitizes PPAR-*γ*
nTZDpa		PPAR-*γ* selective agonist	Anti diabetic, anti carcinogenic
JTT-501 (isoxazolidinedione)		PPAR-*γ* selective agonist	Anti diabetic
Pioglitazone hydrochloride		Selective PPAR-*γ* agonist	Anti diabetic
S26948		Selective PPAR-*γ* agonist	Anti diabetic
Troglitazone		Selective PPAR-*γ* agonist	Anti diabetic
FH535		PPAR-*γ* antagonist	Inhibits Wnt/*β*-catenin signaling
GSK0660		PPAR-*γ* antagonist	Inverse agonist PPAR-*β*/*γ*
GSK3787		PPAR-*γ* antagonist	Inverse agonist PPAR-*β*/*γ*
BADGE		PPAR-*γ* selective	Antagonist for roziglitazone
LG-100641		PPAR-*γ* selective	Blocks TZDs antagonist
